# Vaginal micronized progesterone capsule versus vaginal progesterone gel for lutheal support in normoresponder IVF/ICSI-ET cycles

**DOI:** 10.12669/pjms.312.6613

**Published:** 2015

**Authors:** Kenan Sofuoglu, Ismet Gun, Sadik Sahin, Okan Ozden, Oktay Tosun, Mustafa Eroglu

**Affiliations:** 1 Kenan Sofuoglu, Department of Obstetrics and Gynecology, Zeynep Kamil Training and Education Hospital, Istanbul, Turkey; 2 Ismet Gun, GATA Haydarpaşa Training Hospital, Department of Obstetrics and Gynecology, Istanbul, Turkey; 3 Sadik Sahin, Department of Obstetrics and Gynecology, Zeynep Kamil Training and Education Hospital, Istanbul, Turkey; 4 Okan Ozden, GATA Haydarpaşa Training Hospital, Department of Obstetrics and Gynecology, Istanbul, Turkey; 5 Oktay Tosun, GATA Haydarpaşa Training Hospital, Department of Obstetrics and Gynecology, Istanbul, Turkey; 6 Mustafa Eroglu, Department of Obstetrics and Gynecology, Zeynep Kamil Training and Education Hospital, Istanbul, Turkey

**Keywords:** IVF/ICSI-ET cycles, Luteal Phase Support, Progesterone

## Abstract

**Objective:**

To compare the outcomes of luteal phase support by micronized progesteron vaginal capsule 600mg/day and progesterone vaginal gel 180mg/day in the normoresponder IVF/ICSI-ET cycles of the patients down-regulated via GnRH agonist long protocol or fixed antagonist protocol below 40 years of age.

**Methods:**

A total of 463 normoresponder cycles between January 2013 and December 2013 were retrospectively analyzed. Those with a BMI>28 kg/m^2^, any kind of uterine, ovarian or adnexial pathology, any significant systemic, endocrine or metabolic disease or who were reported as azoospermia, were excluded from the study. The patients were grouped according to the usage of micronized progesterone vaginal capsule 600mg/day (Group 1) or progesterone vaginal gel 180mg/day (Group 2) as luteal phase support. Treatment cycle characteristics and pregnancy outcomes were compared between groups.

**Results:**

Group-I included 220 cycles and group 2 included 243 cycles. Although the MII oocyte percentage among the total number of MII oocytes was significantly higher in Group-II (77.5% and 80.2%; p=0.034), positive ß-hCG (32.3% and 21.8%; p=0.015) and clinical pregnancy (27.3% and 17.7%; p=0.018) rates were significantly higher in Group-I. No difference was observed between groups regarding the ongoing pregnancy rates (23.2% and 17.3%; p=0.143).

**Conclusion:**

Micronized progesterone vaginal capsule 600mg daily used for luteal support in the IVF/ICSI-ET cycles was observed to significantly increase the biochemical and clinical pregnancy rates compared to progesterone vaginal gel 180mg daily. However, no difference was observed between two groups regarding ongoing pregnancy rates.

## INTRODUCTION

The success of in vitro fertilization/intracytoplasmic sperm injection (IVF/ICSI) treatments depends upon many known and unknown factors, the most important being the implantation window. The implantation rates have not yet reached the desired level despite the advancements observed in the field. Oocyte quality and endometrial receptivity factors have important roles in the implantation process.^1^,^2^ It has been reported in many studies and meta-analyses that luteal phase defect (LPD) is encountered following the controlled ovarian hyperstimulation (COH) in both the GnRH agonist and antagonist protocols, and that luteal phase support (LPS) should routinely be administered in these cycles in order to increase the implantation rates.^3^-^7^ However, many subjects including the most proper agent, the timing to start, duration and route of administration are still controversial although the requirement of LPS is proven.^7^-^12^ Many agents and combinations have been used for luteal support until today.^3^,^5^,^7^,^13^,^14^ Progesterone is the most preferred and discussed agent among those.^15^

The aim of this study was to compare the pregnancy outcomes obtained in the IVF/ICSI-ET cycles of the patients with normal ovarian reserve who were given either micronized progesterone vaginal capsule 600mg/day or progesterone vaginal gel 180mg/day for luteal phase support following the COH performed via agonist or antagonist protocols.

## METHODS

This study included the retrospective analysis of the files of the patients who were admitted to the Assisted Reproduction Department of Zeynep Kamil Training and Education Hospital due to the desire of having a child, between January 2013 and December 2013. The study included primary or secondary infertile women with major indications for IVF and who were treated with either conventional agonist long protocol or the GnRH antagonist fixed protocol. Other inclusion criteria were age range of 23-40 years, body mass index of (BMI) 18-28kg/m^2^, regular menstrual cycles between 25 and 35 days, normal basal serum FSH (≤10IU/l) and estradiol (E2) (<75pg/ml) levels measured on the third day of the cycle, fresh cycles, no uterine (fibroids, adenomyosis, mullerian malformations), ovarian (endometrioma, polycystic ovary), or adnexal (hydrosalpinx) pathology assessed by transvaginal ultrasonography. The exclusion criteria were infertility duration longer than 10 years, total gonadotropin dose requirement of more than 4500IU for induction, presence of previous attempts with ≤4 retrieved oocytes, and presence of any significant systemic disease, endocrine, or metabolic disorder. Patients with a diagnosis of azoospermia, those receiving additional estrogen treatment during induction due to thin endometrial echo, those requiring coasting, and cycles that resulted with no embryo for transfer were excluded as well. Datas were obtained from the patient files.

The classical agonist long protocol included the pituitary down-regulation via the subcutaneous administration of triptorelin acetate (Decapeptyl^®^; Ferring Pharmaceuticals A.S.) 0.1mg/day on 21^th^ day of the previous mentrual cycle. GnRH antagonist fixed protocol included direct gonadotropin suppression via the subcutaneous administration of cetrorelix acetate (Cetrotide 0.25mg^®^; Merck Serono) 0.25mg/day on 6^th^ day of the menstrual cycle. Both the GnRH-agonist and GnRH-antagonist protocols continued until the day of hCG administration for ovulation induction. Transvaginal ultrasonography was performed and serum E2 concentration was measured on the 2^nd^ day of the cycle. According to the conventional long protocol, gonadotropin treatment was initiated if no follicles exceeding 10mm in-diameter was observed and the E2 level was below 50pg/ml. According to the antagonist protocol, the gonadotropin treatment was started on the 2^nd^ day of the cycle if no adnexal pathology larger than 20mm in-diameter was observed. The initial gonadotropin dose was determined by the age, BMI, ovarian reserve determined by antral follicle count and basal FSH level of the patients, and experience from the previous cycles. Consecutive ultrasonographic controls and E2 level measurements were performed until three or more follicles with a ≥17 mm diameter and a serum E2 level >500pg/ml were detected. Choriogonadotropin-alpha 250μg (Ovitrelle^®^; Merck Serono, Turkey) was administered subcutaneously (sc.) to induce final follicular maturation. Oocytes were retrieved 35.5 hours after the hCG administration. Fertilization was assessed on the 16^th^ or 18^th^ hours following ICSI procedure and up to two embryos with the best morphological grade, were transferred into the uterine cavity under the guidance of ultrasonography (GE Logic alfa 200). ET was done on day two or three. The policy of our country concerning the number of embryos to be transferred allowed the transfer of 2 embryos in patients older than 35 years of age and those with two or more previous unsuccessful ART cycles. In other circumstances, one embryo was transferred. Biochemical pregnancy was defined as a positive pregnancy test result (hCG levels>20mIU/ml) 12 days after embryo transfer. Clinical pregnancy was defined as fetal cardiac activity observed via vaginal ultrasonography 4 or 5 weeks after oocyte retrieval, and ongoing pregnancy was defined as sonographic control of the embryo after the 9^th^ gestational week.

Luteal support was initiated on the night of oocyte retrieval and continued until the day of pregnancy testing. In order to minimize the potential immune reaction against the transferred embryos, methylprednisolone 16mg/day (Prednol 16mg tablet^®^, Mustafa Nevzat, Turkey) was also given orally to all patients for 4 days. In case the test was positive, progesterone treatment was continued up to 9^th^ gestational weeks.

The patients were categorized in two groups according to the luteal support treatment. Group-I was given micronized progesterone 600mg/day (Progestan^®^ 200mg, soft capsule, Koçak, Turkey) via vaginal route in the form of three equal doses, whereas Group-II was given progesterone 90mg (Crinone %8 gel^®^, Merck Serono, Turkey) via vaginal route twice daily. The groups were compared with regard to their demographic characteristics, induction characteristics and treatment outcomes.

### Statistical analysis

Statistical analyses were performed using the Statistical Package for the Social Sciences for Windows 15.0 software (SPSS, Chicago, IL., USA). Descriptive statistics were given as mean, standard deviation, frequency and percentage. Parametric comparisons were performed using Student’s t-test, and non-parametric comparisons were performed using Mann-Whitney U test. Categorical data were evaluated by using χ^2^ test. Statistical significance was defined as p<0.05.

## RESULTS

A total of 1073 cycles (of 987 patient files) were investigated between January 2013 and December 2013. Among those, 811 cycles included patients with sufficient file information receiving vaginal progesterone for luteal support, 343 of whom employed GnRH agonist long protocol and 468 of whom employed GnRH antagonist fixed protocol. A total of 463 cycles (445 patients) were concluded to be eligible for the study according to the inclusion criteria. Among those, 220 cycles (212 patients) were Group.I and 243 cycles (233 patients) were Group-II. The mean age (± standard deviation) was 31.03±4.27 years (ranged between 24-40) and 46.44% of the indications for IVF were unexplained infertility.

The demographic characteristics of the groups are presented in Table-I. No difference was observed between groups except for age which had no clinical importance (30.51±4.38 and 31.49±4.13, respectively; p=0.013). Table-II shows cycle characteristics and treatment outcomes. Although no difference was detected between groups regarding the total mean number of oocytes and MII oocyte count, MII oocyte percentage among the total oocyte count was statistically significantly higher in Group-II (77.5% and 80.2%, respectively; p=0.034). Consequently, the number of fertilized oocytes was higher in group2 although not significant (4.06±2.49 and 4.22±3.21, respectively; p=0.570). In the contrary, positive ß-hCG rate per cycle was significantly higher in Group-I than Group-II (32.3% and 21.8%, respectively; p=0.015). The same significant difference was observed in favor of group1 among the patients who underwent agonist long protocol (39.8% and 23%, respectively; p=0.016), whereas no difference was observed between groups among the patients who underwent fixed antagonist protocol (27.3% and 20.8, respectively; p=0.277). Similarly, clinical pregnancy rates were significantly higher in Group-I compared to Group-II (27.3% and 17.7%, respectively; p=0.018). However, higher ongoing pregnancy rates in Group-I compared was not statistically significant (23.2% and 17.3%, respectively; p=0.143).

**Table-I:** Demographic characteristics of the groups.

	Group 1 (n=220)	Group 2 (n=243)	P
Age, y	30.51±4.38	31.49±4.13	0.013^a^
Infertility duration, y	4.85±2.29	5.05±2.48	0.385^a^
Antral follicles at day 1, n	14.57±3.56	14.40±3.24	0.584^a^
D3 FSH, IU/l	6.94±1.66	7.17±1.58	0.127^a^
D3 estradiol, pg/ml	43.95±13.41	42.22±15.10	0.196^a^
Infertility diagnosis, n (%)
Tubal factor	33 (15%)	21 (8.64%)	0.047^b^
Male factor	82 (37.27%)	112 (46.09%)	0.068^b^
Mixed	14 (6.36%)	17 (6.99%)	0.932^b^
Unexplained	105 (47.73%)	110 (45.27%)	0.735^b^

Data are presented as mean ± SD and number (percent).

a Student t test.

b χ^2^ test.

**Table-II:** Stimulation characteristics and treatment outcomes of 463 cycles.

	Group 1 (n=220)	Group 2 (n=243)	P
GnRH agonist/antagonist protocol, n	88/132	113/130	0.188^b^
Average used gonadotrophin, IU	2220.66±998.01	2393.92±937.99	0.056^a^
Endometrium on HCG day, mm	10.11±1.69	10.46±1.96	0.055^a^
Serum E2 on HCG day, pg/ml	2305.86±861.67	2334.09±882.71	0.743^a^
Total oocytes retrieved, n	9.55±4.30	9.13±5.07	0.342^a^
MII oocytes, n	7.40±3.63	7.32±4.40	0.835^a^
MII oocytes/total oocytes retrieved, (%)	77.5%	80.2%	0.034^b^
Fertilized oocytes, n	4.06±2.49	4.22±3.21	0.570^b^
ET day, n	2.54±0.50	2.59±0.50	0.260^b^
Embryos transferred, n	1.17±0.37	1.15±0.36	0.642^a^
Positivity of ß-hCG/cycle, (%)	32.3%	21.8%	0.015^b^
GnRH agonist long protocol, n(%)	35/88 (39.8%)	26/113 (23%)	0.016^b^
GnRH antagonist fix protocol, n(%)	36/132 (27.3%)	27/130 (20.8)	0.277^b^
Clinical Pregnancy rate/cycle, (%)	27.3%	17.7%	0.018^b^
GnRH agonist long protocol, n(%)	29/88 (32.9%)	21/113 (18.6%)	0.030^b^
GnRH antagonist fix protocol, n(%)	31/132 (23.5%)	22/130 (16.9%)	0.243^b^
Ongoing pregnancy rate/cycle, (%)	23.2%	17.3%	0.143^b^
GnRH agonist long protocol, n(%)	22/88 (25%)	20/113 (17.7%)	0.277^b^
GnRH antagonist fix protocol, n(%)	29/132 (21.9%)	22/130 (16.9%)	0.243^b^

Data are presented as mean ± SD and number (percent). FSH; follicle stimulating hormone, HCG; human chorionic gonadotropin, E2; estradiole, ET; embryo transfer.

a Student t test.

b χ^2^ test.

## DISCUSSION

It has been known for almost 60 years that LPD is accompanied by poor pregnancy outcomes.^16^ One of the best proofs of this is the study of Csapo et al. where they reported that the surgical excision of corpus luteum led to loss of pregnancy in the early weeks.^17^ Subsequently, this situation was also confirmed with placebo-controlled randomized trials and put forward in two large meta-analyses as well.^3^,^6^ Finally, in their meta-analysis including 16.317 women and investigating the outcomes of LPS involving 69 studies, Van der Linden and et.al revealed, in accordance with the conclusions of 15 clinical trials comparing patients receiving and not receiving progesterone, that LPS was necessary in IVF cycles.^7^ Many mechanisms may have roles in the impairment of the luteal phase in IVF treatment cycles including LH suppressive effect of GnRHs,^18^,^19^ possible early developmental effect of short-term supranormal estrogen and progesterone levels on the endometrium during luteal phase in the induced cycles, aspiration of the granulosa cells during the oocyte pick-up (OPU) procedure,^20^ and the blockage of LH release with the negative feed-back effect of the steroids synthesized secondary to the corpora lutea which are many in number.^13^

Thus far, many agents have been used for luteal support.^3^-^5^,^7^,^13^ The most controversial one among them, and which is still used today, has been progesterone. In our study, progesterone for LPS was started on the night of oocyte retrieval in all patients and continued up until the 9^th^ gestational week in case of pregnancy. However, the minumum amount of progesterone necessary for the maintenance of pregnancy, the most efficacious from of progesterone and whether a treatment in addition to progesterone is necessary or not, are topics of debate. The largest series on the dose of progesterone is found in the meta-analysis done by van der Linden et al. in 2001.^7^ In this meta-analysis, patients groups given ≤100mg (low-dose) and >100mg (high-dose) vaginal progesteron for LPS were compared; and no difference was observed between the groups in terms of clinical pregnancy rates (12 studies, 4973 patients), ongoing pregnancy rates (5 studies, 3034 patients), miscarriage rates (8 studies, 2350 patients) and live birth rates (2 studies, 1485 patients). Besides, along with progesterone for LPS, hCG, estradiol and GnRHa usages are found in literature. Even though it has often been reported that additional hCG usage increases pregnancy rates, it is not much preferred due to the risk of OHSS.^3^,^5^,^6^,^7^

The general approach in IVF treatments for LPS suggests usage of vaginal progesterone due to both its minimal side-effect spectrum and ease of use.^21^ A survey of relevant literature will show that there are few comparative studies on vaginal progesterone formulations.^22^-^29^ And an examination of this literature will also show that, except for two recent large randomized studies,^28^,^29^ these studies are few in number and not capable in general of distinguishing a difference between different doses and components. Moreover, except for the study by Stadtmauer et al^28^., none of these studies includes live birth rates. Like previous studies, our study has two disadvantages as well. One of them is that it is a retrospective study, and the other is that it does not include live birth rates. When the two recent large studies was examined, it was reported that there were no difference, similar to the outcomes of previous studies, in terms of vaginal gel and vaginal ring or tablet groups and in terms of clinical pregnancy rates, ongoing pregnancy rates or live birth rates.^28^,^29^ While Stadtmauer et al.^28^ started LPS treatment on the day of oocyte retrieval, Bergh et al. have started it on the day of embryo transfer. There is only one study in the literature on this subject. Here LPS treatment was started in three different times (hCG day, oocyte retrieval day and embryo transfer day) and no difference was reported between them in terms of ongoing pregnancy rates (20.8%, 22.7% and 23.6%, respectively).^8^

As a conclusion, progesterone appears to be the ideal agent for LPS treatment. According to the results of our study, 600mg/day micronized vaginal progesterone usage for LPS treatment increases positive pregnancy and clinical pregnancy rates in comparison to 180mg/day vaginal gel usage. While this increase was observed in patients undergoing GnRH agonist protocol, it was not observed in patients undergoing GnRH antagonist protocol. Although ongoing pregnancy rates were higher in the micronized vaginal progesterone capsule group, they were not statistically significant. Larger, prospective, multi-centered and randomized studies are needed in order to increase both implantation rates and ongoing pregnancy rates.
